# A Laboratory Critical Incident and Error Reporting System for Experimental Biomedicine

**DOI:** 10.1371/journal.pbio.2000705

**Published:** 2016-12-01

**Authors:** Ulrich Dirnagl, Ingo Przesdzing, Claudia Kurreck, Sebastian Major

**Affiliations:** 1 Department of Experimental Neurology and Center for Stroke Research Berlin (CSB), Charité Universitätsmedizin Berlin, Berlin, Germany; 2 German Center for Neurodegenerative Diseases (DZNE), Berlin, Germany; 3 German Center for Cardiovasular Diseases (DZHK), Berlin, Germany; 4 Excellence Cluster NeuroCure, Berlin, Germany; 5 Berlin Institute of Health, Berlin, Germany

## Abstract

We here propose the implementation of a simple and effective method to enhance the quality of basic and preclinical academic research: critical incident reporting (CIR). CIR has become a standard in clinical medicine but to our knowledge has never been implemented in the context of academic basic research. We provide a simple, free, open-source software tool for implementing a CIR system in research groups, laboratories, or large institutions (LabCIRS). LabCIRS was developed, tested, and implemented in our multidisciplinary and multiprofessional neuroscience research department. It is accepted by all members of the department, has led to the emergence of a mature error culture, and has made the laboratory a safer and more communicative environment. Initial concerns that implementation of such a measure might lead to a “surveillance culture” that would stifle scientific creativity turned out to be unfounded.

“*Learning without thought is labour lost;*

*Thought without learning is perilous*.”

—*Confucius, 551–479 B.C. (cited after* [1]*)*

The realization that only a disappointingly small fraction of preclinical studies can be replicated (“replication crisis” [2–4]) and the exceedingly low rate of preclinically very successful treatment strategies that actually end up benefitting patients (“translational roadblock” [5]) have kindled a discussion questioning the robustness, rigor, and productiveness of current experimental biomedical research. Numerous systematic reviews and meta-analyses in various medical fields have exposed a prevalence of low internal validity of this type of research resulting from bias (such as selection, performance, outcome, and reporting bias) in addition to exceedingly low sample sizes and consequently low statistical power. Alarmed by the waste of resources and the harm to patients this might cause, researchers, journals, funders, and institutions have rushed to investigate, initiate, and implement measures for improving the quality of preclinical research. Not surprisingly, many of these measures are inspired by clinical medicine. Cognizant of deficits in study quality, clinical medicine went through a similar process many decades ago. The subsequent implementation of measures such as blinding and randomization, extensive and standardized record keeping, monitoring and auditing, preregistration of studies, and quality management systems, to name but a few, greatly improved the conduct and the validity of results in clinical research.

As a result of the current discussion relating to nonreproducibility of preclinical biomedical research, a plethora of recommendations and potential remedies have been suggested [6–8]. These include a number of approaches to improve research quality in biomedical laboratories, ranging from the authentication and standardization of biologicals and reagents [9] to the implementation of full-blown quality management systems[10–13]. We here propose a simple and effective method to enhance the quality of preclinical research: critical incident reporting (CIR). CIR has become a standard in clinical medicine, and quality management systems recommend error management (e.g., International Organization for Standardization (ISO) 9001:2008; clause 8.3, “Control of Non-conforming Product,” [14]). To our knowledge, however, CIR has never been implemented in the context of academic basic research. Although there are an increasing number of publications on preclinical quality management (e.g., [6,10–13,15,16]), none of these articles mentions CIR or offers recommendations on how to learn from errors and prevent them in the future. We here provide a simple, free, open-source software tool written in the Python programming language to implement a simple CIR system in research groups, laboratories, or institutions (LabCIRS, S1 Text).

Critical incident monitoring and reporting was initially described in 1954 [17] by Flanagan, who adapted it from techniques developed to improve safety and performance among military pilots in World War II. In clinical medicine, CIR was first introduced in anesthesiology in 1978 [18]. Today, reporting of critical incidents is an internationally recognized tool for improving patient safety in clinical medicine. By introducing it into their legal regulations, many countries have made CIR a mandatory element of hospital safety and quality management procedures. The basic principle of CIR in clinical medicine is that safety can be improved by learning from incidents that could have harmed or did harm patients, rather than by ignoring such incidents. Adverse incidents are recorded anonymously, analyzed, discussed, and communicated so that a recurrence can be prevented and harm avoided by learning from past mistakes. This occurs primarily at the local (departmental or hospital) level, but databases made available via the internet have facilitated the expansion of incident recording in clinical medicine to the national and even international level.

The highly complex setting of a modern biomedical research laboratory with its high-tech machinery, multiprofessional and often international staff with different levels of expertise, complicated assays, and potentially harmful chemicals or biologicals is comparable to that of aviation or human surgery and anesthesia. In preclinical research, critical incidents and errors involve events that have the potential to negatively impact data integrity, experimental outcomes, animal welfare, personnel safety, or viability of expensive reagents or machinery. Errors, mishaps, and mistakes of variable severity frequently occur in this environment. While certain areas like animal welfare or work with genetically modified organisms are strictly regulated, no structured quality management systems are required in preclinical research, and hence, no procedures for error management are mandated. Errors or critical incidents may only be reported sporadically or erratically or might even be covered up for fear of negative consequences [19].

## A Laboratory CIRS for Academic Biomedical Research

In clinical medicine, it is often necessary to clearly define and classify critical incidents. This is not the case in preclinical research, in which there is no standard set of terms for recording such incidents. However, the particularly open nature of the research process may lead to unanticipated and even unorthodox events that deserve reporting. We set out to improve academic biomedical research by systematically learning from errors and mistakes. We established a simple and scalable CIRS suitable for the preclinical biomedical research environment. We then implemented and tested it in a typical, multidisciplinary academic laboratory. The Department of Experimental Neurology, with approximately 100 students, researchers, and technicians, carries out multiprofessional academic research in preclinical biomedicine with such standard approaches and techniques as in vivo and in vitro modeling of disease, cell biology, molecular biology, and biochemistry, as well as imaging (from multiphoton microscopy to magnetic resonance imaging).

Box 1 lists the essential features we expect from a CIRS in basic and preclinical biomedicine.

**Box 1. Features of a Laboratory CIR System for Experimental Biomedicine**

- Easy to set up, run, and administer
- Easy to use, accessible, intuitive, and unambiguous
- Should be scalable so that it works in small single-investigator groups as well as in a large institute
- Allows anonymous reporting
- Allows free expression of “what actually happened”: the reporter’s own version of events
- Reports must be handled in a nonpunitive manner
- The incidents reported can be regularly analyzed by experts
- Learning points from such analyses need to be fed back promptly to those who need to know
- The reports are visible, and a clear path of action is communicated
- Feedback results in enhanced learning regarding the incident’s cause and systemic changes that will prevent its recurrence

**Fig 1:**
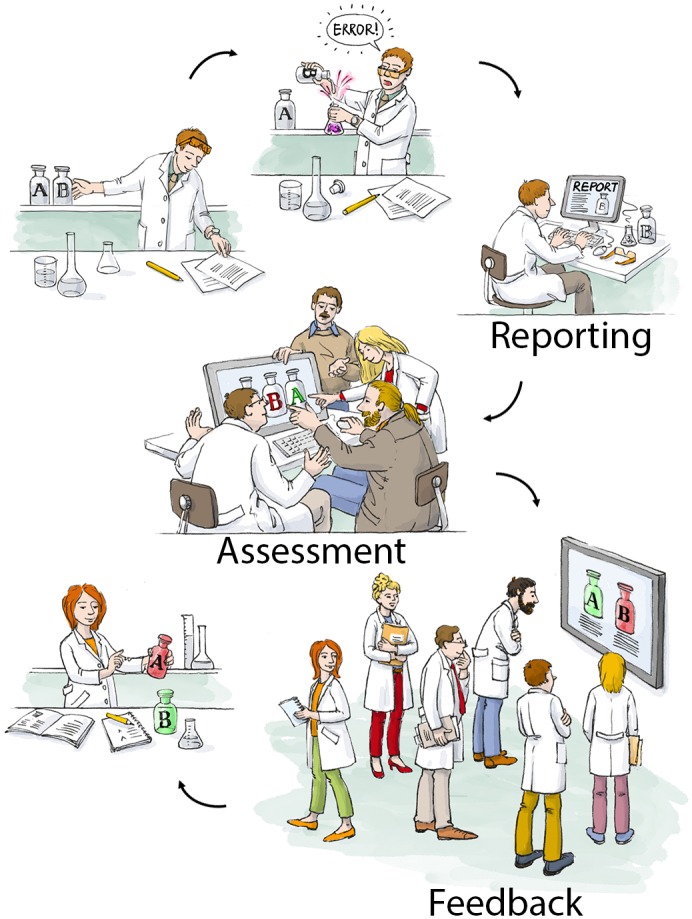
Cartoon of how LabCIRS helps to prevent further mishaps and fosters an error culture. “Error”: a researcher mistook two faintly labeled reagents A and B, which ruined his experiment. “Reporting”: entry of the incident into LabCIRS. “Assessment”: a group of experts (scientists and technicians) reviews the error and takes preventive action by color labelling the reagents. “Feedback”: the errors as well as the measure to prevent it in the future are communicated to the entire laboratory.

The flow of information and resultant activities in LabCIRS is summarized in Fig 1:

LabCIRS can be accessed from every computer logged into the intranet of the department. Incidents are reported anonymously, either in German or in English (see S1 and S2 Figs). A demo version is accessible at http://labcirs.charite.de (sign in as “reporter”). The LabCIRS “reviewer,” who could be a principal investigator (PI), a lab manager, or any other person with the skills to initially assess reports, is alerted to incoming reports via email. The reviewer assigns a risk category (low to high), determines responsibilities, initiates subsequent measures, and decides who is responsible for their implementation (see S3 Fig, sign in as “reviewer” in the demo version). All reported incidents are analyzed in a regular monthly quality assurance conference. Depending on the nature of the reported incident, additional expert members of the department may be invited to join the discussion. Time critical events are processed immediately. Agreement is reached on specific prevention measures, responsibilities, and an action plan. Relevant preventive measures are communicated to all members of the department at the weekly morning conferences, and a monthly email to all staff members summarizes events and countermeasures. These messages are accessible to everyone and are permanently archived in the LabCIRS. Errors and incidents are communicated anonymously, unless the incident report includes the name of the reporting person and consent to reveal his or her identity. Typically, the examples of incidents reported via the LabCIRS include injuries when working with a sharp object, mistaken labeling of solutions and chemicals, mix-ups in the randomization of experimental animals, and data loss due to instrument write failures.

## Emergence of an Error Culture

Motivated by the exceedingly high attrition rate of bench to bedside translation in the stroke research field, we began to establish a structured quality management (QM) system in our experimental laboratories in 2012. The aim of our QM system is to implement auditable standards for the planning, realization, evaluation, and publication of our experimental studies and to safeguard compliance to guidelines (such as [20]) and institutional rules and regulations of good scientific practice (GSP). We realized that for academic preclinical research there is a paucity, if not to say a lack, of systematic approaches to improve and maintain quality. We had to therefore design from scratch, implement, and refine effective and transparent procedures for quality control in experimental neuroscience research in the university setting. To the best of our knowledge, our QM system represents one of the first attempts to implement systematic QM in academic preclinical research in Germany and possibly worldwide.

Since the ISO 9001:2008 norm [14], which we chose for our QM, requires the implementation of measures for identifying errors, we posted printed “error reporting sheets” in all our laboratories. Disappointingly, only a few errors were reported via this mode. Through discussions with scientists, students, and technicians, we realized that the main reason for the failure of this error reporting system was that it did not safeguard anonymous reporting; potential reporters feared punitive action. We therefore established the web-based system described here, which includes additional benefits, such as accessibility on every computer in the lab, uploading of photographs to describe the incident, automatic alerts of new reports to personnel responsible, and archiving.

As most academic research laboratories operate on a frugal budget, and funding organizations might be reluctant to cover costs beyond specific research projects, it is important to know how resource intense the operation of LabCIRS is. In our department when an error is reported, the reviewer categorizes the incident and decides whether acute measures are necessary. If the reporter agreed to it, the report is communicated to the department. Error reports are analyzed in detail at monthly meetings by a group consisting of various users and experts (scientists, doctoral students, postdocs, and technicians) of the research groups. The group also considers preventive measures against recurrence of the error. Relevant errors and countermeasures are then communicated to all members of the department in a weekly joint lab meeting. Additionally, all error reports are sent out monthly to all members of the department via email. All in all, analyzing, discussing, and communicating one error report takes about 40 minutes.

**Fig 2:**
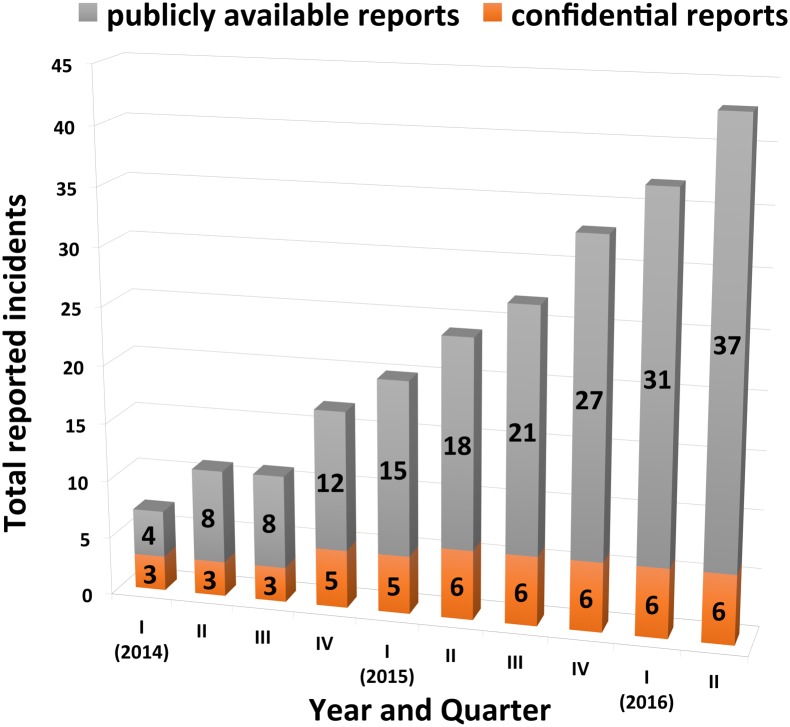
Errors reported per quarter since system was initiated (I/2014) until June 2016. Grey: number of errors reported publicly; orange: number of errors reported confidentially, i.e., without allowing the report to be made public.

LabCIRS was immediately accepted by all members of the laboratory. Since its inception, approximately one to two incidents are reported per month (Fig 2). Interestingly, in the beginning about half the reported incidents were not only anonymous but also strictly confidential (i.e., the reporters ticked the option “I DO NOT agree that this report will be made public to people outside the quality management team even after copyedit.”). For more than a year now, all reporters tick the option “I agree that this report will be made public to people outside the quality management team after copyedit.” We interpret this as a sign of trust and an indicator of a mature error culture. Clearly, most members of the department have realized that while in the complex setting of the biomedical laboratory, errors and incidents may occur, they can be prevented in future through reflection on what happened and through the input of colleagues. All reported errors have led to actions and preventive measures. These include modifications of briefings, instructions, and responsibilities, changes in the way samples and chemicals are labelled, and modifications of standard operating procedures, among many other provisions.

**Fig 3:**
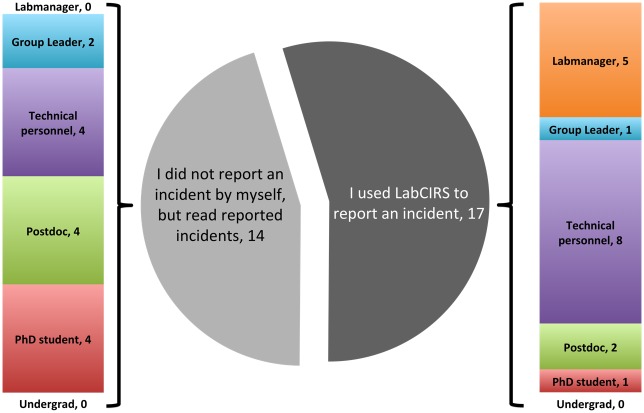
Results of an anonymous survey to explore which professions and status groups use LabCIRS in an active or passive manner.

Anonymous reporting is a key feature of any CIRS. LabCIRS does not collect any personal information from the reporter, as such information in a relatively small group of approximately 100 people could potentially reveal the identity of the reporter. In order to nevertheless address the question of whether the use of LabCIRS differs between professions, we conducted an anonymous online survey asking two questions: (1) Do you actively and/or passively use LabCIRS (i.e., have you reported errors, or do you only read about errors in LabCIRS)? and (2) What is your job profile or status within the lab? About half of the responding LabCIRS users stated that they actively report incidents, while the other half uses the CIRS to stay informed about reported errors and countermeasures. The largest groups of the active reporters are either technicians or lab managers, while students, postdocs, technicians, and group leaders are represented almost equally among the passive users (Fig 3). It is not surprising that the majority of active reporters belong to the groups which focus on practical laboratory work, but our survey demonstrates that members of all professions use LabCIRS either actively or at least passively. The only exception is undergraduate students, who do not work unsupervised in our institute and are guided directly by members of other professions.

We are convinced that LabCIRS has clearly improved the quality of our work and made the laboratory a safer and more communicative environment. Although desirable, it is unfortunately hard to quantify the effectiveness of such a measure. The use of a CIRS cannot be tested in a controlled experiment (one lab with and the other without CIRS). CIRS use is voluntary and anonymous, and reported incidents do not necessarily represent all incidents that may have happened. It should be noted that in many domains the use of CIRS is plausible and by now the legal standard (aviation, nuclear power plants, etc.), but rarely, if at all, has its efficiency been unequivocally proven in a controlled setting. Therefore, the efficacy of CIRS must often remain anecdotal, much like the notion that the Chernobyl disaster could have been prevented by critical incidence reporting [21].

Initial concerns by some members of the department that its implementation might lead to a “surveillance culture” that would stifle creativity turned out to be totally unfounded. Nevertheless, it needs to be acknowledged that reporting mistakes, mishaps, and errors is a sensitive issue in any work environment. Setting up a critical incidence reporting system must rely on an intense communication among all members of the lab about its purpose and nonpunitive nature. In addition, reporting of an incident is only the beginning of a sequence of events that include the search for remedies and preventive measures. This only works in a collaborative and quality-oriented environment. However, the administrative effort to maintain such a system is minimal and likely compensated by the savings made through error prevention and improved quality.

We highly recommend the establishment of a systematic way of learning from errors and mistakes, whether in small single-investigator groups of a few researchers, students, and technicians, or in large research institutions with staffs of several hundred professionals. This practice will benefit the emergence of an error culture that will likely enhance the overall quality and safety of research. The open-source LabCIRS we provide here can help to start this process, but it needs to be stressed that the system lives with those who report, discuss, and disseminate the incidents and countermeasures.

## Supporting Information

S1 Fig Screenshot of LabCIRS login. (TIF)

S2 Fig Screenshot of LabCIRS incident reporting page. (TIF)

S3 Fig Screenshot of LabCIRS incident reviewer login. (TIF)

S1 Text Demo version and source code. (DOCX)

## Abbreviations

CIR: critical incident reporting
GSP: good scientific practice
ISO: International Organization for Standardization
LabCIRS: laboratory critical incident reporting system
PI: principal investigator
QM: quality management
