# Supplementary figures and images for: A Laboratory Critical Incident and Error Reporting System for Experimental Biomedicine
Source: PLoS Biol. 2016 Dec 1;14(12):e2000705. doi: 10.1371/journal.pbio.2000705 (PMC5131907; doi:10.1371/journal.pbio.2000705)

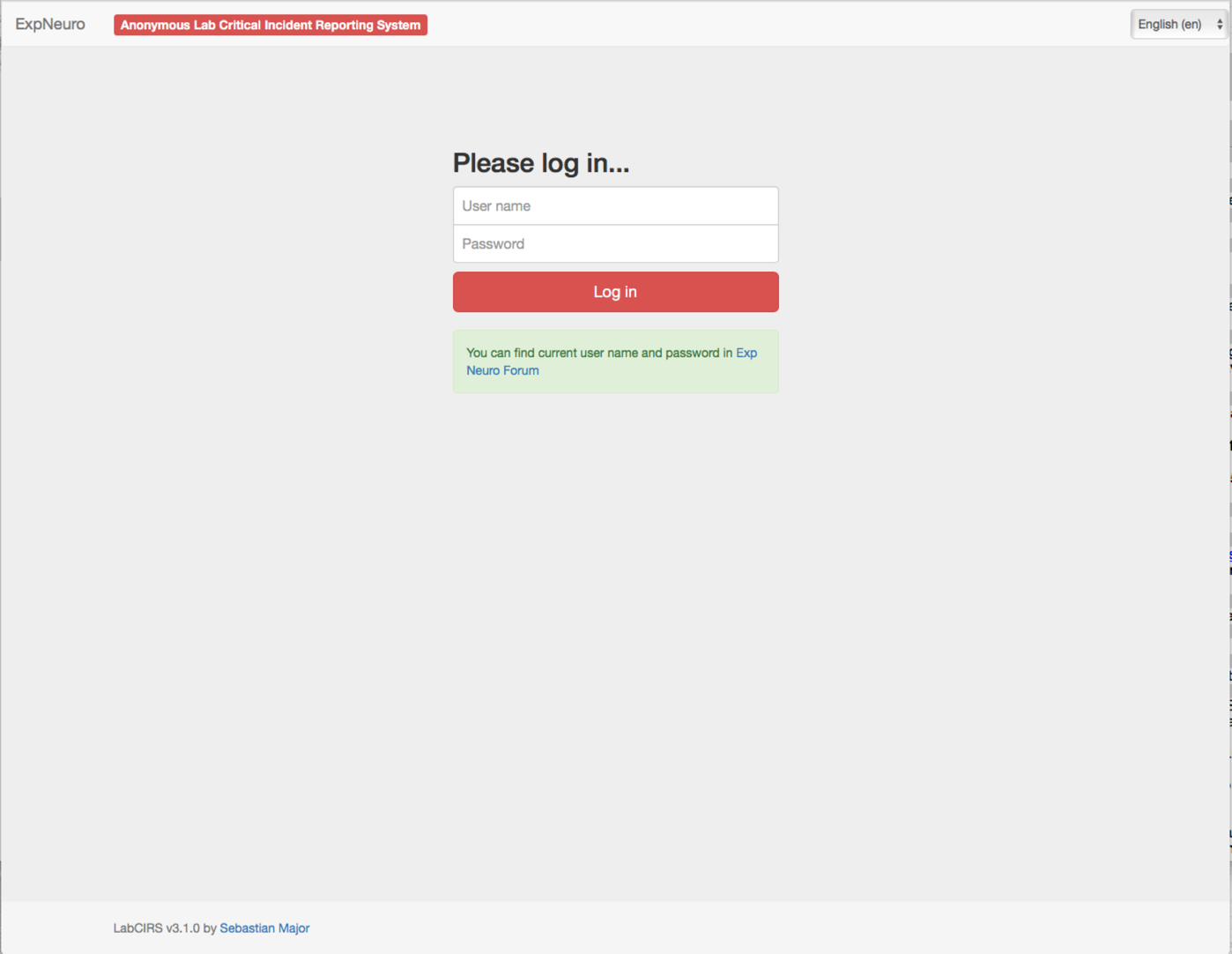

Supplement: S1 Fig — (TIF) [file pbio.2000705.s001.tif]

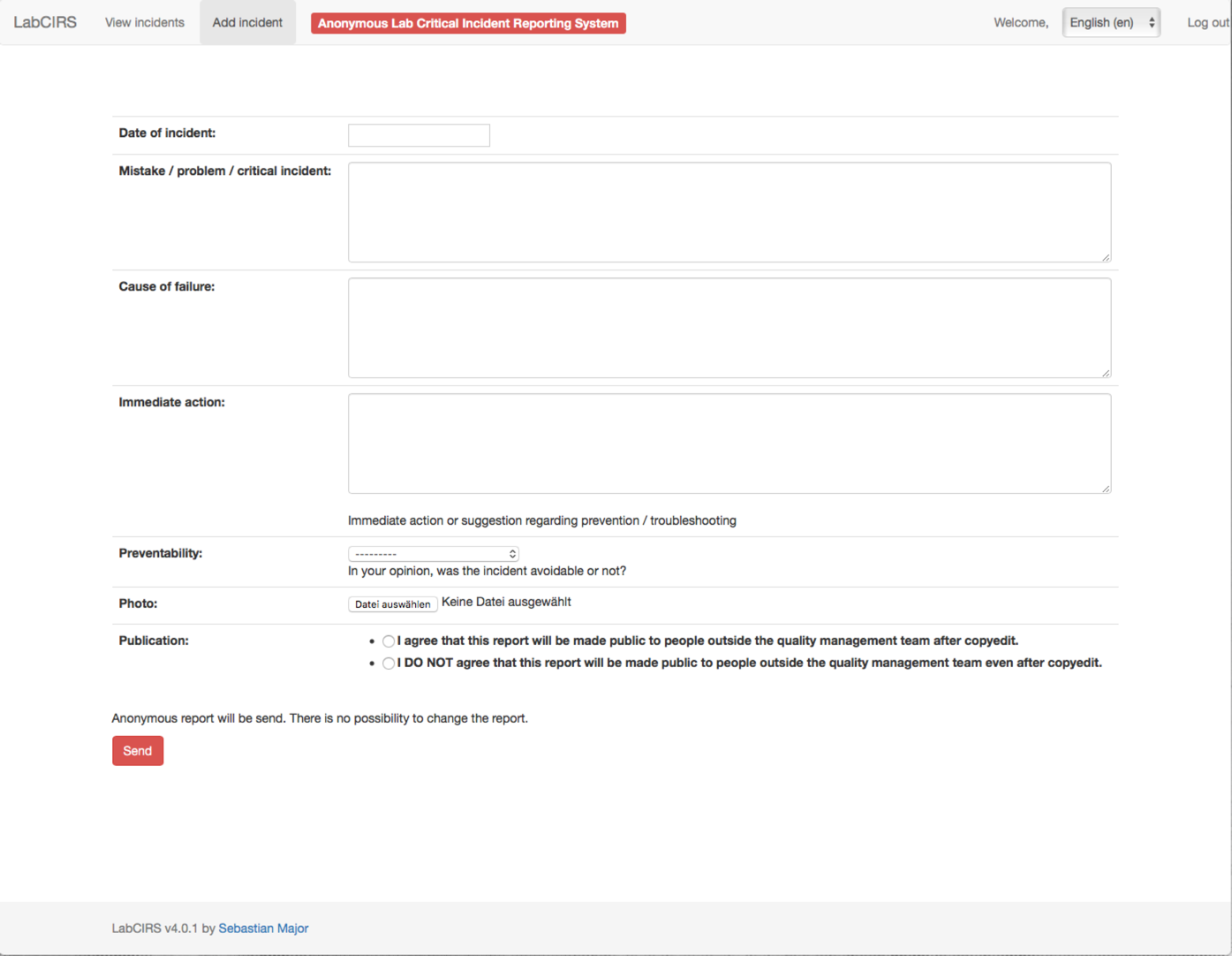

Supplement: S2 Fig — (TIF) [file pbio.2000705.s002.tif]

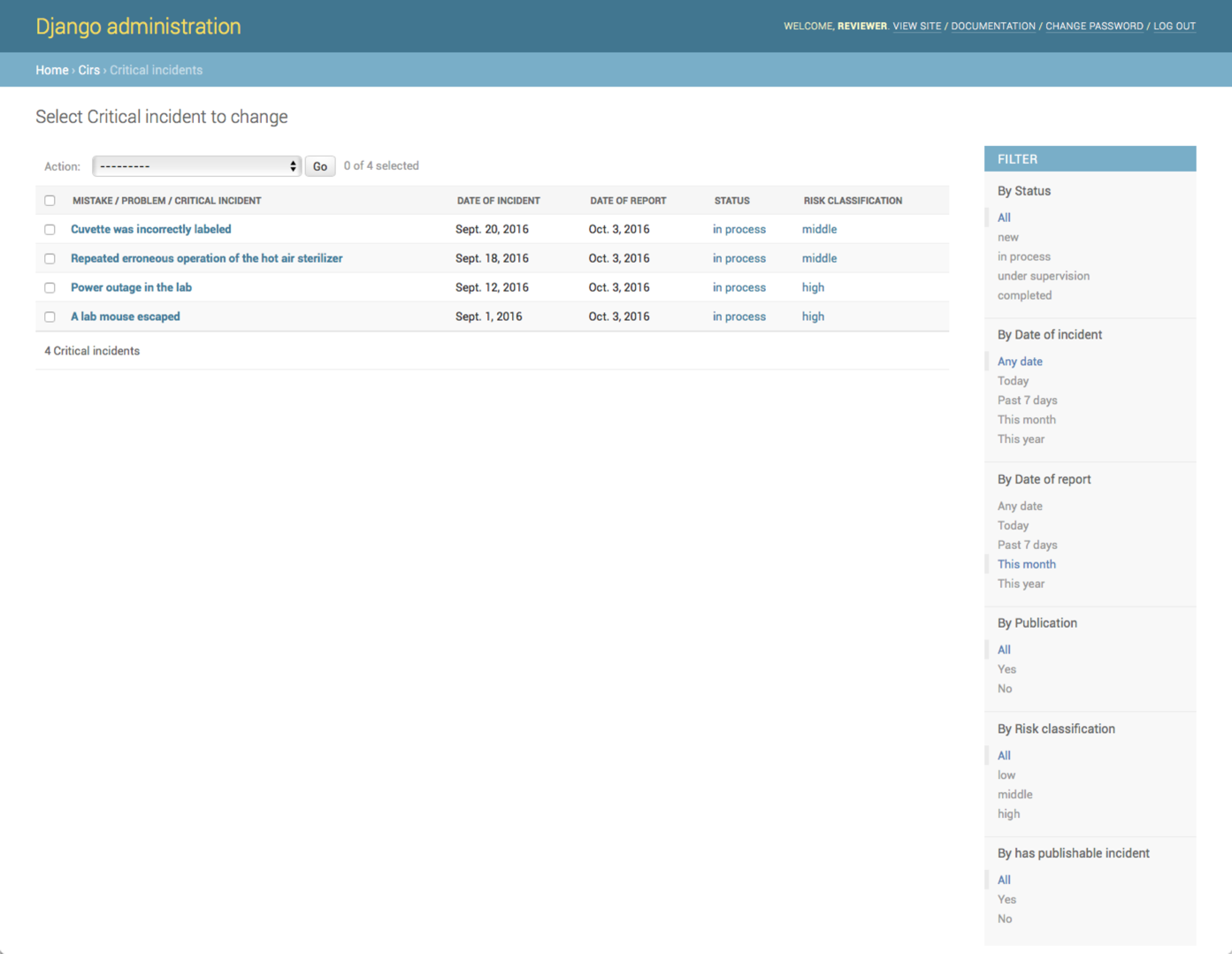

Supplement: S3 Fig — (TIF) [file pbio.2000705.s003.tif]
