# Supplementary material for: A Laboratory Critical Incident and Error Reporting System for Experimental Biomedicine
Source: PLoS Biol. 2016 Dec 1;14(12):e2000705. doi: 10.1371/journal.pbio.2000705 (PMC5131907; doi:10.1371/journal.pbio.2000705)
Supplement: S1 Text — (DOCX) [file pbio.2000705.s004.docx]

Supplementary material 1: Demoversion and source code

A **demo version** of LabCIRS can be accessed at <http://labcirs.charite.de>

You can log in either as a regular user and simulate the reporting of an incident (‘Reporter’), or as administrator reviewing incidents (‘Reviewer’)

**LabCIRS source code** can be downloaded at GITHUB: <https://github.com/major-s/labcirs>

LabCIRS is written in the Python programming language and builds upon Django, an open-source web framework (<http://www.djangoproject.com>). It is free software provided under the GNU General Public License (GNU GPLv2) which allows [end users](https://en.wikipedia.org/wiki/End_user) (individuals, organizations, companies) to run, study, share (copy), and modify the software.

The LabCIRS front-end used for incidence reporting was written from scratch. The user interface adapted to mobile devices as well as to desktop computers was implemented with the widely used Cascading Style Sheets (CSS) framework Bootstrap (http://getbootstrap.com). The backend for reviewing and copy-editing of reported incidents utilizes Django’s built-in administration interface. Internalization of both components is possible. An English or a German user interface can be chosen, implementation of further languages is planned. As web application, LabCIRS can be deployed to a variety of operating systems. It only requires a Webserver Gateway Interface (WSGI) capable web server (e.g. Apache with mod_wsgi) and any database system which is compatible with Django, like MySQL, PostgreSQL, Oracle or even SQLite. Application reliability was probed with several tests, covering approximately 90% of the Python source code.
